# Mass-fractal growth in niobia/silsesquioxane mixtures: a small-angle X-ray scattering study

**DOI:** 10.1107/S1600576714017105

**Published:** 2014-09-04

**Authors:** Rogier Besselink, Johan E. ten Elshof

**Affiliations:** a MESA+ Institute for Nanotechnology, University of Twente, PO Box 217, Enschede 7500 AE, The Netherlands

**Keywords:** mass-fractal growth, niobia, silsesquioxane, small-angle X-ray scattering

## Abstract

The nucleation and growth of niobium pentaethoxide (NPE)-derived clusters in ethanol, through acid-catalyzed hydrolysis/condensation in the presence and absence of the silsesquioxane 1,2-bis(triethoxysilyl)ethane (BTESE), was monitored at 298–333 K by small-angle X-ray scattering. The data were analyzed with a newly derived model for polydisperse mass-fractal-like structures. At 298–313 K in the absence of BTESE the data indicated the development of relatively monodisperse NPE-derived structures with self-preserving polydispersity during growth. The growth exponent was consistent with irreversible diffusion-limited cluster agglomeration. At 333 K the growth exponent was characteristic for fast-gelling reaction-limited cluster agglomeration. The reaction yielded substantially higher degrees of polydispersity. In the presence of BTESE the growth exponents were substantially smaller. The smaller growth exponent in this case is not consistent with irreversible Smoluchowski-type agglomeration. Instead, reversible Lifshitz–Slyozov-type agglomeration seems to be more consistent with the experimental data.

## Abstract 2

The nucleation and growth of niobium pentaethoxide-derived clusters in ethanol was monitored at 298–333 K by small-angle X-ray scattering. The data were analyzed with a newly derived model for polydisperse mass-fractal-like structures.

## 1. Introduction   

Condensed organically bridged silsesquioxanes exhibit substantially higher hydrothermal, acid and mechanical stability than SiO_2_ (Oliver *et al.*, 2010 ▶; Castricum, Sah *et al.*, 2008 ▶; Castricum, Kreiter *et al.*, 2008 ▶; Kanezashi *et al.*, 2010 ▶; Agirre *et al.*, 2014 ▶). Silsesquioxane-based materials can therefore withstand very harsh conditions, which makes them potentially suitable as carriers for catalytic materials. Silsesquioxane-based materials can also exhibit micropores, when processed properly, suitable for filtration and pervaporation or for membrane reactors (Castricum, Sah *et al.*, 2008 ▶; Castricum, Kreiter *et al.*, 2008 ▶; Kanezashi *et al.*, 2010 ▶; Agirre *et al.*, 2014 ▶, 2011 ▶; Kreiter *et al.*, 2011 ▶). To broaden the application window of these hybrid materials to other molecular separation processes and heterogeneous catalysis, bridged silsesquioxanes may be modified by homogeneously doping them with transition metal oxides. The resulting transition metal centers may be useful as catalytically active sites and/or to alter the molecular diffusivity and/or adsorption of molecules in these materials. For instance, the introduction of nickel, cobalt, zirconium and niobium oxides in silica and hybrid silica membranes was found to enhance the H_2_/N_2_ and H_2_/CO_2_ permselectivity (Igi *et al.*, 2008 ▶; Kanezashi & Asaeda, 2006 ▶; Qi *et al.*, 2010 ▶, 2012 ▶; Boffa *et al.*, 2008 ▶, 2009 ▶; Yoshida *et al.*, 2001 ▶). Nevertheless, homogeneous distribution of metal oxides within a matrix of bridged silsesquioxanes is not straightforward from a synthetic point of view. The metal oxide is typically introduced either as a metal salt or as a metal alkoxide that tends to phase separate while the network forms. Metal salts do not contribute to the gel network of the silsesquioxane and are therefore not compatible. Metal alkoxides do form gels; however, their reactivity towards water is orders of magnitude higher than that of silicon alkoxides (Livage *et al.*, 1988 ▶; Kessler *et al.*, 2006 ▶; Brinker, 1990 ▶).

Much is known about the hydrolysis and condensation kinetics of silicon alkoxides *via* ^29^Si-NMR (Sanchez & McCormick, 1992 ▶; Rankin & McCormick, 2000 ▶; Assink & Kay, 1993 ▶). NMR cannot be used for metal alkoxides, because of the very fast kinetics of these materials (Livage *et al.*, 1988 ▶; Kessler *et al.*, 2006 ▶; Brinker, 1990 ▶) and the complex nature of their quadrupolar nuclei (Bonhomme *et al.*, 2007 ▶). Only a limited number of kinetic studies on metal alkoxides have been reported, which rely on techniques including small-angle X-ray scattering (SAXS), Fourier transform infrared spectroscopy, electron conductivity and dynamic light scattering (Hu *et al.*, 2000 ▶; Harris *et al.*, 1997 ▶; Simonsen & Søgaard, 2010 ▶; Broussous *et al.*, 2002 ▶; Wilkinson *et al.*, 1994 ▶; Fan *et al.*, 2006 ▶). Alternatively, macroscopic phenomena including turbidity and gel time have been used to predict effects on the mol­ecular level (Percy *et al.*, 1999 ▶; Sedlar & Sayer, 1995 ▶; Ranjit & Klabunde, 2005 ▶). However, the kinetics on the microscopic level are not always directly correlated with the kinetics on the molecular level (Kessler *et al.*, 2006 ▶).

The extremely fast early-stage reaction kinetics of titanium and zirconium alkoxides have already been described in the work of Harris *et al.* (1997 ▶). In the work presented here, we focus on the nucleation and growth of mixed niobium pentaethoxide (NPE)–1,2-bis(triethoxysilyl)ethane (BTESE) sol–gels in ethanol. Incorporation of niobium into silica or organically modified silica has proven to be useful for CO_2_ scavenging membranes and Lewis acid catalysts (Boffa *et al.*, 2008 ▶; Tanabe, 1990 ▶; Francisco *et al.*, 2004 ▶; Yoshida *et al.*, 1996 ▶). Our aim was to study the earliest stages of growth in these ethanol mixtures, since these sols determine the degree to which niobium is incorporated into a BTESE matrix on the atomic scale. The kinetics of the reaction were monitored by time-resolved synchrotron SAXS experiments. This approach allowed us to determine not only the agglomerate sizes but also their shape and polydispersity in 5 s time frames.

## 2. Experimental   

### 2.1. Sol preparation   

All precursor solutions were prepared in a glove box under nitrogen atmosphere and atmospheric pressure. Firstly, a mixed metal alkoxide precursor solution was made in dry ethanol, which contained 0.635 mol l^−1^ BTESE sol and 0.318 mol l^−1^ NPE (ABCR, Karlsruhe, Germany, 99.99%). This corresponds to an [Si]:[Nb] ratio of 4:1 and an overall ethoxide concentration [–OEt] = 5.4 mol l^−1^, *i.e.* including the ethoxide groups of both BTESE and NPE. Secondly, diluted nitric acid solutions with [H_2_O] = 3.857 mol l^−1^ and [HNO_3_] = 0.129 mol l^−1^ in dry ethanol were prepared. By mixing an equal volume of the combined alkoxide precursor solution and the diluted nitric acid solution, a hydrolysis ratio [H_2_O]/[(Si)–OEt] = 1, an acid ratio [HNO_3_]/[(Si)–OEt] = 1/30 and [–OEt] = 2.7 mol l^−1^ was reached. Particle growth in these solutions was monitored at 298, 313 and 333 K, and these samples are hereafter referred to as SiNbT298, SiNbT313 and SiNbT333, respectively.

Experiments were also performed in the absence of BTESE. NPE precursor solutions with a concentration of 0.318 mol l^−1^ Nb were prepared. Similar to the mixtures with BTESE, exactly the same diluted nitric acid solutions were used, mixed in equal volume ratio with the NPE precursor solution. Particle growth in these mixtures was also monitored at 298, 313 and 333 K. These samples are hereafter referred to as NbT298, NbT313 and NbT333, respectively.

### 2.2. Mixing setup   

**Figure 1:**
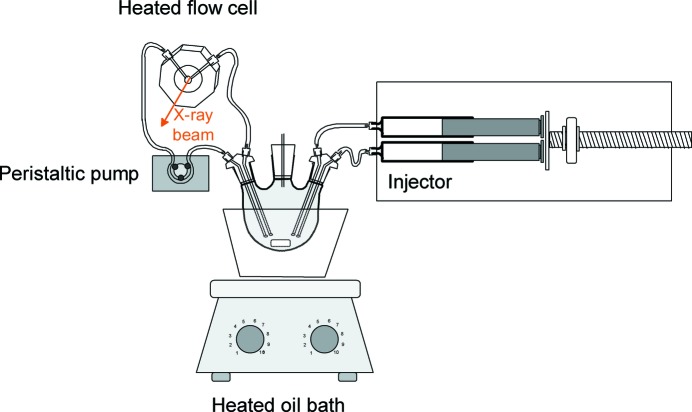
Schematic diagram of mixing setup used in SAXS experiments.

SAXS mixing experiments were executed with a setup as illustrated in Fig. 1 ▶. A homemade flow cell was used, which consisted of an aluminium housing with Luer lock connectors for connecting tubes. The cell comprised two Kapton foils that were compressed against an aluminium interior by two aluminium rings with sealing rubbers outside the cell. The cell itself was cylindrical with a depth of 1 mm and a cross-sectional diameter of 5 mm. Continuous flow between the flow cell and the reactor, which consisted of a 25 ml three-necked round-bottom flask, was arranged by a Masterflex peristaltic pump. The dead time between the round-bottom flask and the flow cell was 10 s. SAXS data collection was started before mixing the solutions, to ensure that data were recorded immediately upon mixing the solutions. The measurement was initiated by a timer that started the injector. In 8 s the injector injected both 12 ml of diluted nitric acid solution and 12 ml of metal alkoxide solution simultaneously into the three-necked round-bottom flask. The round-bottom flask contained a small vent to release overpressure that was formed during injection.

### 2.3. Time-resolved SAXS experiments   

Small-angle X-ray scattering was carried out using synchrotron radiation on the Dutch–Belgian beamline, DUBBLE BM-26B, of the ESRF in Grenoble (Bras *et al.*, 2003 ▶). The X-ray beam with an energy of 12 keV was focused on a corner of the two-dimensional Pilatus 1M pixel detector to maximize the covered range of scattering angles. The detector was placed at a distance of 1.36 m from the sample, which resulted in a measurable scattering vector magnitude (*q*) range 0.17 < *q* < 6.00 nm^−1^. All scattering data were found to be independent of the azimuthal angle in the plane of the detector, which indicated that particles were isotropic or isotropically dispersed throughout the sample. Therefore, all channels with the same *q* value were averaged with each other. Silver behenate was used as calibration standard for the determination of the absolute scale of *q* in our experiments (Bras *et al.*, 2004 ▶). All curves were normalized by dividing the scattering intensity by the signal of the ionization chamber in front of the sample. A background subtraction procedure was carried out. For sols, the scattering signal of a capillary filled with ethanol was subtracted.

## 3. Characterization of SAXS data   

### 3.1. Mass-fractal agglomerates with an exponential cutoff length ξ   

After mixing of components, small clusters of BTESE and NPE will form by hydrolysis and condensation and subsequently grow and agglomerate into amorphous assemblies of primary clusters after a short period of time. These agglomerates have been described in terms of mass fractals in previous work (Besselink *et al.*, 2013 ▶). There, we did not observe a clear transition from the fractal regime (intensity *I* ∝ *q* ^−*D*_f_^) to the Porod regime (*I* ∝ *q* ^−4^), where *D*_f_ is the so-called fractal dimension of the scattering entities. Therefore, the size and the nature of the primary nuclei that build up the agglomerate could not be determined accurately. For the sake of simplicity, these primary nuclei were assumed to be infinitely small. In such a case the pair correlation function is described by (Sorensen & Wang, 1999 ▶)
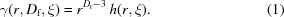
Here, *r* is an isotropic distance in real space and *h*(*r*, ξ) is a cutoff function that defines the finite size of an agglomerate as characterized by the cutoff distance ξ. The exponential cutoff function *h*(*r*, ξ) = exp(−*r*/ξ) is mostly used, and consequently the rotationally averaged Fourier transform of equation (1) results in (Sorensen & Wang, 1999 ▶)
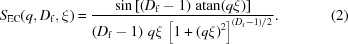
However, most SAXS curves reveal a sharper transition from the Guinier region to the mass-fractal region [equation (2)]. Consequently, a sharper cutoff function would give a more realistic representation. It is convenient to define a function of which the cutoff behavior can be related to the degree of polydispersity. To this end, we first introduce an infinitely sharp cutoff function, *i.e.* a unit step or Heaviside step function *h*(*r*, ξ) = *H*(ξ − *r*). Secondly, polydispersity is introduced by the integral
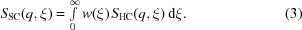
Here, *w*(ξ) is an intensity-weighted probability density function of the cutoff parameter ξ and *S*_HC_ is the rotationally averaged Fourier transform of *H*(ξ − *r*). We selected a Schultz–Zimm distribution function (Kotlarchyk & Chen, 1983 ▶), which has been found to give realistic results in earlier studies on similar sol–gel systems (Pontoni *et al.*, 2002 ▶; Stawski *et al.*, 2011*a* ▶,*b* ▶; Besselink *et al.*, 2013 ▶):
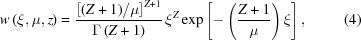
where μ is the intensity-weighted average of ξ and the *Z* parameter is related to the distribution of the cutoff distance, *i.e.* the variance of ξ corresponds to (σ_ξ_)^2^ = μ^2^/(*Z* + 1). The integral of equation (3) can be solved for integer values of *Z* and can be approximated for fractional values of *Z*, as explained in more detail in the supporting information.^1^ As shown there, the scattering intensity can be described by the following equation:
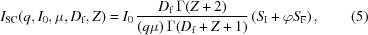
where
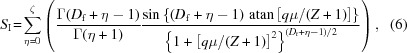


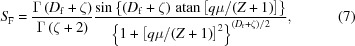
ζ = floor(*Z*) and ϕ = *Z* − ζ.

**Figure 2:**
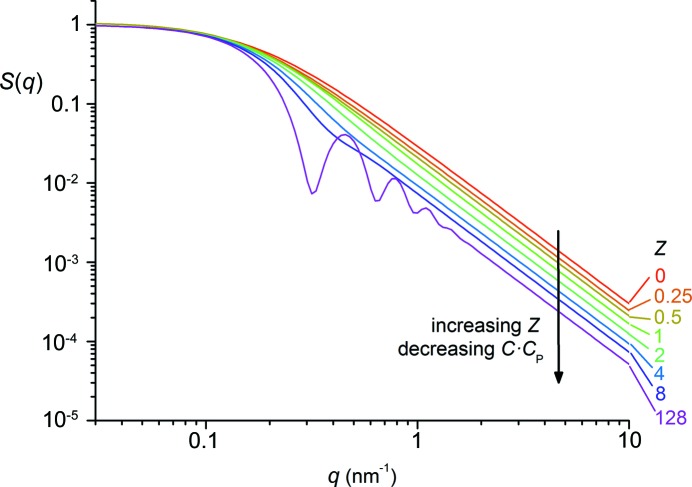
Simulated curves of *I*_SC_(*q*, *I*_0_, μ, *D*_f_, *Z*), equation (5), with *I*_0_ = 1, *R*_G_ = 10 nm and *D*_f_ = 2 being held constant and *Z* = 0, 0.25, 0.5, 1, 2, 4, 8, 128. Note that *I*_SC_(*q*) = *S*_SC_(*q*) since *I*_0_ = 1.

This equation depends only on four independent parameters, namely *I*_0_, μ, *D*_f_ and *Z*. The parameter ζ is the integer part of *Z*, ϕ is the fractional part of *Z*, and η is an integer that is used in the Riemann sum and varies from 0 to ζ. A decreasing polydispersity is accompanied by an increasing *Z* value, which results in a sharper cutoff in the SAXS curves, as illustrated by the simulations of equations (5)–(7) [Disp-formula fd7] in Fig. 2 ▶. Instead of μ we can also calculate the radius of gyration *R*_G_ as a measure of the particle size:
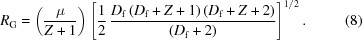
See the supporting information for a derivation of equation (8).

### 3.2. Growth kinetics   

After a very short nucleation period, growth is presumably accomplished by irreversible coagulation of primary nuclei, which can by described by Smoluchowski-type agglomeration kernels (Pontoni *et al.*, 2002 ▶; Oh & Sorensen, 1997 ▶; Pierce *et al.*, 2006 ▶; Ball *et al.*, 1987 ▶; Wu & Friedlander, 1993 ▶):
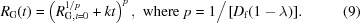
Here, *t* is the reaction time, *p* is a growth exponent that determines the order of the reaction, *k* is a reaction rate constant and λ is the homogeneity parameter. This equation includes a crossover regime between diffusion-limited cluster aggregation (DLCA) (λ ≃ 0) and reaction-limited cluster aggregation (RLCA) (λ ≃ 1) mechanisms and is only valid for non-gelling agglomeration kernels (λ < 1). For gelling agglomeration kernels where λ > 1, equation (9) is no longer valid and particle growth is characterized by (Ball *et al.*, 1987 ▶; van Dongen & Ernst, 1985 ▶)
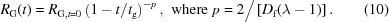
Here *t*_g_ is the gel time. These agglomeration kernels imply a self-preserving polydispersity. This means that depending on the growth-limiting process (*i.e.* to what extent it is described by either DLCA or RLCA) a particular extent of polydispersity is preserved throughout the reaction. The relationship between *D*_f_, λ and the polydispersity parameter *C*_P_ depends on the aggregation regime as determined by the Knudsen number (Kn) (Pierce *et al.*, 2006 ▶). Owing to interaction with solvent molecules, in liquid media the mean-free path of a particle is extremely small and Kn ≃ 0. DLCA growth processes typically lead to structures with *D*_f_ ≃ 1.9, while RLCA leads to higher values (Boffa *et al.*, 2009 ▶). Particles move by diffusion following the Stokes–Einstein relationship with a diffusion coefficient *D* ∝ *R*_h_^−1^, where *R*_h_ is the hydrodynamic radius of a particle. In such a case *C*_P_ is described by (Sorensen & Wang, 1999 ▶)
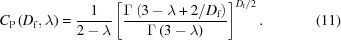



## 4. Results and discussion   

### 4.1. Agglomeration of NPE in the absence of BTESE   

**Figure 3:**
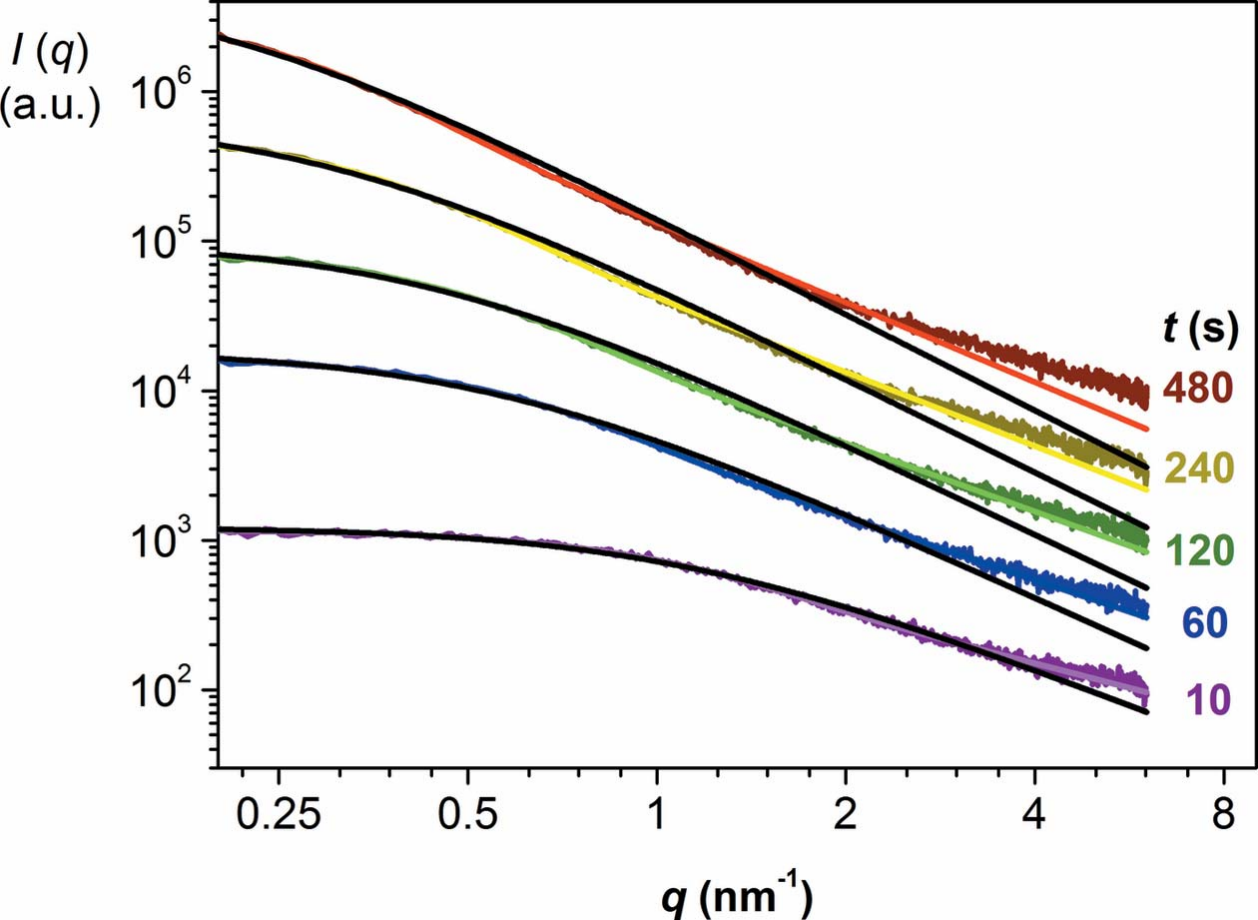
Fitting experimental data of sample NbT298 at different time intervals by using either the exponential cutoff model (black lines) or the Schultz cutoff model (colored lines).

As Fig. 3 ▶ (colored lines) illustrates, the time-resolved experiments on sample NbT298 can be described well by the Schultz cutoff function (5). The exponential cutoff function [equation (2), black lines in Fig. 3 ▶] does not describe the sharp curvature of the experimental data in the Guinier region properly. Consequently, the *D* and *R*_G_ values obtained from the latter fits are systematically larger than those obtained from the Schultz cutoff function. For instance, for *t* = 60 s with the Schultz cutoff, *Z =* 6.3 (7), *D*_f_ = 1.39 (1) and *R*_G_ = 2.51 (7) nm, while for the exponential cutoff *D*_f_ = 1.93 (4) and *R*_G_ = 2.92 (7) nm. In view of the goodness of fit to these experimental data, the Schultz cutoff model was considered more suitable for these data sets and used in the further analyses.

**Figure 4:**
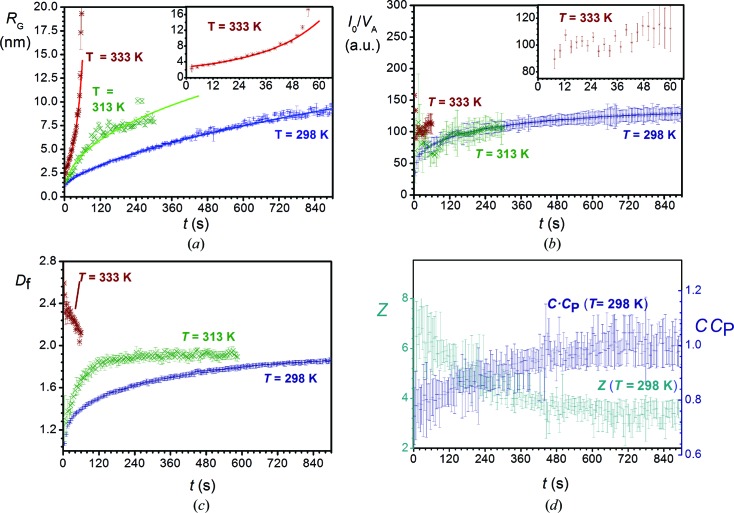
Optimized parameters derived from fitting the Schultz cutoff function for mixing experiments without BTESE. (*a*) *R*_G_, (*b*) *I*_0_/*V*_A_ and (*c*) *D*_f_ at *T* = 298, 313 and 333 K, and (*d*) *Z* and *CC*_P_ at *T* = 298 K.

Upon mixing the NPE solutions and diluted nitric acid solutions in the absence of BTESE (samples NbT298, NbT313 and NbT333), small agglomerates with *R*_G_ > 1 nm were formed instantaneously (Fig. 4 ▶ *a*). For sample NbT333, *R*_G_ increased as *R*_G_ ∝ *t* ^3.8^ (Table 1 ▶), which is consistent with a gelling agglomeration kernel as described by equation (10). The corresponding sol became turbid after 80 s and gelled visually after approximately 180 s. At lower temperatures (298–313 K), particles grew roughly following *R*_G_ ∝ *t* ^0.5^, being consistent with non-gelling agglomeration kernels, even though these sols also gelled eventually, after 1 d and 2 h for NbT298 and NbT313, respectively. Their growth rate can be described by equation (9). The optimized fit parameters are listed in Table 1 ▶.

The ratio *I*_0_/*V*_A_ (Fig. 4 ▶ *b*), which is proportional to the mass of agglomerates in the sol (where *V*_A_ is the average agglomerate volume; equation (S7) of the supporting information), was used as a measure of the extent of particle nucleation. At both 298 and 313 K roughly 50% of the total mass nucleated before the first SAXS data points were recorded. *I*_0_/*V*_A_ leveled off after 120 s, while *I*_0_/*V*_A_ leveled off after *t =* 12 s at 333 K. Beyond these points, the mass concentration of agglomerates was nearly constant and growth occurred predominantly through cluster–cluster aggregation. Note that the data collection for 313 and 333 K was stopped when *R*_G_ became too large. Consequently, *I*_0_ and *V*_A_, which are both coupled to *R*_G_, could not be determined with sufficient accuracy either.

In the initial stages of the reactions, the number of primary particles present in the agglomerate is too small for the agglomerate to be regarded as a mass fractal. In the course of time, an increasing number of primary particles contributed to the agglomerate until, eventually, well defined mass fractals evolved. In the case of sample NbT298 at *t* = 480 s, the fractal region extended over the range 0.4 < *q* < 6 nm^−1^ (Fig. 3 ▶) and a mass fractal dimension could be roughly established. The value of *D*_f_ leveled off towards *D*_f_ = 1.88 (Table 1 ▶), as illustrated in Fig. 4 ▶(*c*). At 313 K the particles grew substantially faster: the rate constant *k* was about eight times larger than that at 298 K, while the growth exponent *p* was roughly 1.2 times smaller (Table 1 ▶). Effectively, the agglomerates grew four times faster at 313 K than at 298 K. *R*_G_ reached ∼6 nm after 120 s of growth, while at 298 K a similar gyration radius was reached after *t* = 480 s. Consequently, *D*_f_ leveled off to *D*_f_ = 1.91 after only 120 s of reaction time at 313 K. Owing to the limited statistics of *D*_f_, the decreasing trend within the first 60 s at *T* = 333 K may not be significant, but the value of *D*_f_ was clearly larger than the values found at lower temperatures.

At 298 K a decreasing value of *Z* and an increasing value of *CC*_P_ (where *C* is a constant related to the geometry of the fractal agglomerate) within the first 480 s imply increasing polydispersity within this time frame. After *t* = 480 s both values leveled off. This trend can be explained by the extent of nucleation as characterized by the ratio *I*_0_/*V*_A_. As long as new primary particles nucleate while other agglomerates grow, the polydispersity will continue to increase. However, as the ratio *I*_0_/*V*_P_ levels off, *Z* and *CC*_P_ level off as well. This is consistent with agglomeration kernels with a self-preserving particle distribution, *i.e.* during growth of fractal-like agglomerates when the mass concentration (∼*I*_0_/*V*_P_) is being preserved (Sorensen & Wang, 1999 ▶; Oh & Sorensen, 1997 ▶; Pierce *et al.*, 2006 ▶; Wu & Friedlander, 1993 ▶; van Dongen & Ernst, 1985 ▶; Dekkers & Friedlander, 2002 ▶). For the sake of clarity the trends of *Z* and *CC*_P_ at 313 K were omitted from the figure since they strongly overlap with the data at 298 K. Nevertheless, throughout the reaction their values remained almost constant within the error margins of the fit. Optimized values are listed in Table 1 ▶. At *T* = 333 K, *Z* and *CC*_P_ remained constant at 0 and 3.0, respectively, indicating a substantially higher degree of polydispersity as compared to agglomeration at 298 and 313 K.

At both 298 and 313 K the early-stage agglomeration process is probably a non-gelling DLCA mechanism, as is suggested by the final values *D*_f_ ≃ 1.9 (Boffa *et al.*, 2009 ▶) and *CC*_P_ ≃ 1.0. The growth exponent *p* was consistent with λ close to zero, which further confirms the proposed DLCA mechanism. Yet, the *C*_P_ value calculated from λ was ∼1.5, larger than the static *C*_P_ values of ∼1.0 as derived from *Z*. Since *C* is related to the geometry of a single agglomerate, it is possibly smaller in the present case (*C* ≃ 2/3) than in previously reported experimental data (Sorensen & Wang, 1999 ▶). Our findings suggest a lower variation in branch sizes per agglomerate, *i.e.* agglomerates that are less elongated and more spherically shaped.

At 333 K, agglomeration was consistent with a gelling reaction-limited (cluster) aggregation RL(C)A mechanism, as indicated by *D*_f_ ≃ 2.5, *CC*_P_ ≃ 3.0 and λ ≃ 1.3 (Table 1 ▶). Between 313 and 333 K, the mechanism changed from a well controlled DLCA mechanism with a low degree of polydispersity at 313 K to a poorly controlled RL(C)A mechanism with a high degree of polydispersity at 333 K.

### 4.2. Agglomeration of NPE/BTESE mixtures   

**Figure 5:**
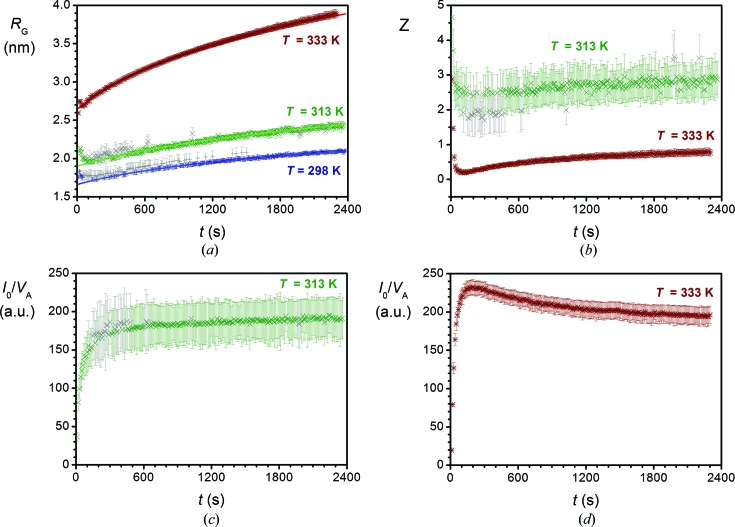
Optimized parameters derived from fitting the Schultz cutoff function for mixing experiments with BTESE. (*a*) *R*_G_ at *T* = 298, 313 and 333 K, (*b*) *Z* at *T* = 313 and 333 K, (*c*) *I*_0_/*V*_A_ at *T* = 313 K, and (*d*) *I*_0_/*V*_A_ at *T* = 333 K.

The agglomeration of sol–gel mixtures that contained both BTESE and NPE (samples SiNbT298, SiNbT313 and SiNbT333) was characterized in terms of the parameters *Z*, *R*_G_ and the ratio *I*_0_/*V*_A_ (Fig. 5 ▶). After a very short induction period, the *Z* parameter remained constant at *Z* ≃ 2.5 at 313 K (see Fig. 5 ▶ *b*, colored symbols). Especially in the early stages of the reaction, when the size range of the fractal regime is very limited, deviations of the *Z* parameter were observed. Moreover, owing to the relatively short time, measurements may also be sensitive to thermal noise. For the sake of clarity the data of *Z* at 298 K are not shown in Fig. 5 ▶(*a*), since they strongly overlap with the very similar trend of *Z* at 313 K. At 333 K, *Z* decreased more rapidly to *Z* ≃ 0.19, and in analogy with agglomeration in the absence of BTESE, particles became more polydisperse as compared to agglomeration at 298 and 313 K. However, *Z* slowly increased after reaching a minimum value after 120 s (*Z* = 0.19) and subsequently leveled off at *Z* = 0.77. Thus, the degree of polydispersity decreased upon prolonged growth.

At all temperatures, *R*_G_ increased most rapidly in the first 10 s after mixing (Fig. 5 ▶ *a*). After the first 10 s, *R*_G_ increased slowly by 25% at 298 and 313 K, and by 40% at 333 K. By comparing data from experiments with and without BTESE (Tables 1 ▶ and 2 ▶, and Figs. 4 ▶ and 5 ▶), it can be concluded that the presence of BTESE had a negligible effect on the initial size of the nuclei. However, subsequent growth of niobia clusters was clearly suppressed by the presence of BTESE. The growth exponent *p* was substantially smaller when BTESE was present, which leads to unrealistic values of λ when it is assumed that agglomeration can be described by irreversible Smoluchowski-type rate equations. Instead, the reduced growth exponents *p* were more consistent with reversible Lifshitz–Slyozov-type agglomeration kernels (Pontoni *et al.*, 2002 ▶; Lifshitz & Slyozov, 1961 ▶; Conti *et al.*, 2004 ▶; Sharon *et al.*, 2003 ▶). This also holds at 333 K, where the process was characterized as a fast-gelling agglomeration kernel in the absence of BTESE. Most likely, the nucleated niobia particles/clusters were surrounded by BTESE-derived moieties, which affected their agglomeration behavior.

In analogy with the experiments without BTESE, nucleation occurred predominantly within the first 120 s as indicated by the ratio *I*_0_/*V*_A_ *versus* time (Fig. 5 ▶ *c* and 5 ▶ *d*). However, the values of *I*_0_/*V*_A_ were substantially larger; they included the nuclei from both NPE and BTESE precursors. Within a short nucleation period at 298 and 313 K, *Z* leveled off (Fig. 5 ▶ *b*) even more quickly than *I*_0_/*V*_A_. In the subsequent growth process the extent of polydispersity was preserved. Compared to the experiments without BTESE, both *Z* and *D*_f_ leveled off to lower values, which effectively resulted in a similar value for *CC*_P_.

At 333 K (Fig. 5 ▶ *d*), *I*_0_/*V*_A_ increased very rapidly within the first 120 s, then increased further to a maximum at *t* = 200 s. After the maximum it slowly descended and leveled off at 83% of its maximum value. Because *I*_0_/*V*_A_ ∝ *N* *V*_A_ is proportional to a volume-weighted average volume (Feigin & Svergun, 1987 ▶; Porod, 1982 ▶) (*i.e. V*_A_ = 〈*V* ^2^〉/〈*V*〉, where *V* includes the series of all particle volumes being measured at a given time), its value increases with increasing polydispersity at a constant mass concentration of particles since its value is dominated by the largest particles in the system. This could explain the decreasing value of *I*_0_/*V*_A_ upon subsequent growth, while *Z* increased and polydispersity decreased. The decrease in polydispersity may be explained by Oswald ripening, which would be consistent with reversible Lifshitz–Slyozov-type agglomeration processes.

Especially at 333 K, the trends of *Z* and *I*_0_/*V*_A_ imply two separate processes: (1) a fast and poorly controlled irreversible nucleation process yielding highly polydisperse particles, which is followed by (2) a slow reversible Lifshitz–Slyozov-type cluster–cluster agglomeration process that allows Oswald ripening. Only the second, reversible, stage of the process was clearly affected by the presence of BTESE, and this stage can be associated with BTESE condensation, while the first growth stage predominantly involved irreversible agglomeration of NPE into small nuclei. This confirms that the phase-separated niobia domains that were found in the dried xerogel (Besselink *et al.*, 2013 ▶) were formed instantaneously upon hydrolyzing the sol. Moreover, both in the absence and in the presence of BTESE a clear difference was found between agglomerations at 333 K as compared to 298–313 K. At 333 K the initial agglomeration of niobia clusters was reaction limited, which resulted in a substantially higher degree of polydispersity than after agglomeration of niobia clusters at 298 and 313 K.

## 5. Conclusions   

At *T* = 298 and 333 K the condensation and agglomeration of NPE can be described by diffusion-limited cluster aggregation, as supported by the homogeneity parameter λ ≃ 0. At *T* = 333 K the same system gels and the value λ ≃ 1.28 is consistent with a gelling reaction-limited aggregation mechanism. The polydispersity as indicated by the *Z* parameter increased when the temperature was increased from 313 to 333 K.

The rate of agglomeration *k* and the growth exponent *p* in BTESE/NPE mixtures were clearly suppressed as compared to the NPE mixtures without BTESE. The low *p* values for BTESE/NPE mixtures are more consistent with reversible agglomeration processes. In analogy with the mixtures without BTESE, the polydispersity as indicated by the *Z* parameter increased substantially when the temperature was increased from 313 to 333 K.

## Supplementary Material

Theory of mass-fractal agglomerates with an exponential cut-off length. DOI: 10.1107/S1600576714017105/vh5007sup1.pdf

## Figures and Tables

**Table 1:** Characteristic parameters of the self-preserving Smoluchovski-type reaction kernels of fractalic particles with a Schultz cutoff function in the absence of BTESE

Parameter	NbT298	NbT313	NbT333
*R*_G,*t* = 0_	1.22 (2)	0	2.68 (6)
*p*	0.56 (1)	0.44 (2)	3.8 (1)
*k* (s^−1^)	0.060 (2)	0.50 (1)	–
*t*_g_ (s)	–	–	170 (30)

*D*_final_	1.88 (1)	1.91 (4)	2.45 (2)
*Z*_final_	3.2 (6)	4.8 (16)	0.0 (1)
*CC*_P_final_(*Z*, *D*_f_)	1.0 (1)	0.9 (2)	3.0 (1)

λ	0.04 (1)	−0.20 (5)	1.28 (1)
*C*_P_(λ, *D*_f_)	1.5 (1)	1.5 (1)	2.4 (1)

**Table 2:** Characteristic parameters of the self-preserving Smoluchovski-type reaction kernels of fractalic particles with a Schultz cutoff function in the presence of BTESE

Parameter	SiNbT298	SiNbT313	SiNbT333
*R*_G,*t* = 0_	1.66 (4)	1.92 (1)	2.64 (1)
*p*	0.14 (2)	0.18 (2)	0.21 (1)
*k* (s^−1^)	0.061 (1)	0.045 (8)	0.21 (2)

*D*_final_	1.68 (1)	1.66 (2)	1.90 (1)
*Z*_final_	2.58 (13)	2.84 (3)	0.77 (3)
*CC*_P_final_(*Z*, *D*_f_)	1.1 (1)	1.0 (1)	1.79 (2)

## Footnotes

1 Supporting information for this article is available from the IUCr electronic archives (Reference: VH5007).

## References

[bb1] Agirre, I., Arias, P. L., Castricum, H. L., Creatore, M., ten Elshof, J. E., Paradis, G. G., Ngamou, P. H., van Veen, H. M. & Vente, J. F. (2014). *Sep. Purif. Technol.* **121**, 2–12.

[bb2] Agirre, I., Güemez, M., van Veen, H., Motelica, A., Vente, J. & Arias, P. (2011). *J. Membr. Sci.* **371**, 179–188.

[bb3] Assink, R. A. & Kay, B. D. (1993). *Colloids Surf. A Pysicochem. Eng. Asp.* **74**, 1–5.

[bb4] Ball, R. C., Weitz, D. A., Witten, T. A. & Leyvraz, F. (1987). *Phys. Rev. Lett.* **58**, 274–277. 10.1103/PhysRevLett.58.274 10034887

[bb5] Besselink, R., Stawski, T. M., Castricum, H. L. & ten Elshof, J. E. (2013). *J. Colloid Interface Sci.* **404**, 24–35. 10.1016/j.jcis.2013.04.031 23688717

[bb6] Boffa, V., ten Elshof, J., Garcia, R. & Blank, D. (2009). *Microporous Mesoporous Mater.* **118**, 202–209.

[bb7] Boffa, V., ten Elshof, J. E., Petukhov, A. V. & Blank, D. H. (2008). *ChemSusChem*, **1**, 437–443. 10.1002/cssc.200700165 18702139

[bb8] Bonhomme, C., Coelho, C., Baccile, N., Gervais, C., Azaïs, T. & Babonneau, F. (2007). *Acc. Chem. Res.* **40**, 738–746. 10.1021/ar600030j 17461543

[bb9] Bras, W., Dolbnya, I. P., Detollenaere, D., van Tol, R., Malfois, M., Greaves, G. N., Ryan, A. J. & Heeley, E. (2003). *J. Appl. Cryst.* **36**, 791–794.

[bb10] Bras, W., Emsley, J. W., Levine, Y. K., Luckhurst, G. R., Seddon, J. M. & Timimi, B. A. (2004). *J. Chem. Phys.* **121**, 4397–4413. 10.1063/1.1776116 15332991

[bb11] Brinker, C. J. S. G. W. (1990). *Sol Gel Science, The Physics and Chemistry of Sol-Gel Processing.* San Diego: Acedemic Press.

[bb12] Broussous, L., Santilli, C. V., Pulcinelli, S. H. & Craievich, A. F. (2002). *J. Phys. Chem. B*, **106**, 2855–2860.

[bb13] Castricum, H. L., Kreiter, R., van Veen, H. M., Blank, D. H., Vente, J. F. & ten Elshof, J. E. (2008). *J. Membr. Sci.* **324**, 111–118.

[bb14] Castricum, H. L., Sah, A., Kreiter, R., Blank, D. H. A., Vente, J. F. & ten Elshof, J. E. (2008). *J. Mater. Chem.* **18**, 2150–2158. 10.1039/b718082a 18292904

[bb16] Conti, M., Lipshtat, A. & Meerson, B. (2004). *Phys. Rev. E*, **69**, 031406. 10.1103/PhysRevE.69.031406 15089293

[bb17] Dekkers, P. J. & Friedlander, S. K. (2002). *J. Colloid Interface Sci.* **248**, 295–305. 10.1006/jcis.2002.8212 16290534

[bb18] Dongen, P. G. van & Ernst, M. H. (1985). *Phys. Rev. Lett.* **54**, 1396–1399. 10.1103/PhysRevLett.54.1396 10031021

[bb19] Fan, J., Boettcher, S. W. & Stucky, G. D. (2006). *Chem. Mater.* **18**, 6391–6396.

[bb20] Feigin, L. A. & Svergun, D. U. (1987). *Struncture Analysis by Small Angle X-ray and Neutron Scattering.* New York: Plenum Press.

[bb21] Francisco, M. S. P., Landers, R. & Gushikem, Y. (2004). *J. Solid State Chem.* **177**, 2432–2439.

[bb22] Harris, M. T., Singhal, A., Look, J. L., Smith-Kristensen, J. R., Lin, J. S. & Toth, L. M. (1997). *J. Sol-Gel Sci. Technol.* **8**, 41–47.

[bb23] Hu, M. Z., Zielke, J. T., Byers, C. H., Lin, J. S. & Harris, M. T. (2000). *J. Mater. Sci.* **35**, 1957–1971.

[bb24] Igi, R., Yoshioka, T., Ikuhara, Y. H., Iwamoto, Y. & Tsuru, T. (2008). *J. Am. Ceram. Soc.* **91**, 2975–2981.

[bb25] Kanezashi, M. & Asaeda, M. (2006). *J. Membr. Sci.* **271**, 86–93.

[bb26] Kanezashi, M., Yada, K., Yoshioka, T. & Tsuru, T. (2010). *J. Membr. Sci.* **348**, 310–318.

[bb27] Kessler, V. G., Spijksma, G. I., Seisenbaeva, G. A., Håkansson, S., Blank, D. H. A. & Bouwmeester, H. J. M. (2006). *J. Sol-Gel Sci. Technol.* **40**, 163–179.

[bb28] Kotlarchyk, M. & Chen, S. (1983). *J. Chem. Phys.* **79**, 2461–2469.

[bb29] Kreiter, R., Rietkerk, M. D. A., Castricum, H. L., van Veen, H. M., ten Elshof, J. E. & Vente, J. F. (2011). *J. Sol-Gel Sci. Technol.* **57**, 245–252.

[bb30] Lifshitz, I. & Slyozov, V. (1961). *J. Phys. Chem. Solids*, **19**, 35–50.

[bb31] Livage, J., Henry, M. & Sanchez, C. (1988). *Prog. Solid State Chem.* **18**, 259–341.

[bb32] Oh, C. & Sorensen, C. (1997). *J. Aerosol Sci.* **28**, 937–957.

[bb33] Oliver, M. S., Dubois, G., Sherwood, M., Gage, D. M. & Dauskardt, R. H. (2010). *Adv. Funct. Mater.* **20**, 2884–2892.

[bb34] Percy, M., Bartlett, J., Woolfrey, J. & West, B. (1999). *J. Mater. Chem.* **9**, 499–505.

[bb35] Pierce, F., Sorensen, C. & Chakrabarti, A. (2006). *Phys. Rev. E*, **74**, 021411. 10.1103/PhysRevE.74.021411 17025429

[bb36] Pontoni, D., Narayanan, T. & Rennie, A. R. (2002). *Langmuir*, **18**, 56–59.

[bb37] Porod, G. (1982). *Small Angle X-ray Scattering.* New York: Acedamic Press.

[bb38] Qi, H., Chen, H., Li, L., Zhu, G. & Xu, N. (2012). *J. Membr. Sci.* **421–422**, 190–200.

[bb39] Qi, H., Han, J., Xu, N. & Bouwmeester, H. J. (2010). *ChemSusChem*, **3**, 1375–1378. 10.1002/cssc.201000242 20941786

[bb40] Ranjit, K. T. & Klabunde, K. J. (2005). *Chem. Mater.* **17**, 65–73.

[bb41] Rankin, S. E. & McCormick, A. V. (2000). *Chem. Eng. Sci.* **55**, 1955–1967.

[bb42] Sanchez, J. & McCormick, A. (1992). *J. Phys. Chem.* **96**, 8973–8979.

[bb43] Sedlar, M. & Sayer, M. (1995). *J. Sol-Gel Sci. Technol.* **5**, 27–40.

[bb44] Sharon, E., Moore, M. G., McCormick, W. D. & Swinney, H. L. (2003). *Phys. Rev. Lett.* **91**, 205504. 10.1103/PhysRevLett.91.205504 14683375

[bb45] Simonsen, M. E. & Søgaard, E. G. (2010). *J. Sol-Gel Sci. Technol.* **53**, 485–497.

[bb46] Sorensen, C. & Wang, G. (1999). *Phys. Rev. E Stat. Phys. Plasmas Fluids*, **60**, 7143–7148. 10.1103/physreve.60.7143 11970655

[bb47] Stawski, T. M., Veldhuis, S. A., Besselink, R., Castricum, H. L., Portale, G., Blank, D. H. A. & ten Elshof, J. E. (2011*a*). *J. Phys. Chem. C*, **115**, 20449–20459.

[bb48] Stawski, T. M., Veldhuis, S. A., Besselink, R., Castricum, H. L., Portale, G., Blank, D. H. A. & ten Elshof, J. E. (2011*b*). *J. Phys. Chem. C*, **115**, 24028.

[bb49] Tanabe, K. (1990). *Catal. Today*, **8**, 1–11.

[bb51] Wilkinson, A. P., Speck, J. S., Cheetham, A. K., Natarajan, S. & Thomas, J. M. (1994). *Chem. Mater.* **6**, 750–754.

[bb52] Wu, M. & Friedlander, S. (1993). *J. Aerosol Sci.* **24**, 273–282.

[bb53] Yoshida, K., Hirano, Y., Fujii, H., Tsuru, T. & Asaeda, M. (2001). *J. Chem. Eng. Jpn*, **34**, 523–530.

[bb54] Yoshida, H., Tanaka, T., Yoshida, T., Funabiki, T. & Yoshida, S. (1996). *Catal. Today*, **28**, 79–89.

