# Supplementary material for: Mass-fractal growth in niobia/silsesquioxane mixtures: a small-angle X-ray scattering study
Source: J Appl Crystallogr. 2014 Sep 4;47(Pt 5):1606–13. doi: 10.1107/S1600576714017105 (PMC4180739; doi:10.1107/S1600576714017105)
Supplement: Supplementary file 1 [file j-47-01606-sup1.pdf]

Supporting information

Mass-fractal growth in niobia/silsesquioxane mixtures: A SAXS study

R. Besselink,[1] *J.E. ten Elshof*[*1]

[1] Inorganic Materials Science , MESA[+] Institute for Nanotechnology, University of Twente, P.O. Box 217, 7500 AE Enschede, The Netherlands.

[*]Email: j.e.tenelshof@utwente.nl, Tel: +31 53 4892695, Fax: +31 53 4892990

1. Mass-fractal agglomerates with an exponential cut-off length ξ

The diffusion limited cluster aggregation (DLCA) mechanism typically leads to less polydisperse agglomerates, as implied by the exponentially decaying cut-off function (Sorensen & Wang, 1999). Sharper cut-off functions such as a Gaussian cutoff ($h(r,\xi)$= exp(-($r/\xi)^2$) are realistic for a variety of aggregation mechanisms. It would be convenient to define a function of which the cutoff behavior can be related to the degree of polydispersity. To this end, firstly we introduce an infinitely sharp cut-off function, i.e. a unit step or Heaviside step function $h(r,\xi)= H(\xi-r)$. The intensity function of a mass fractal with a hard cutoff function was described by a rotationally averaged Fourier transform:

$$S_{\mathrm{HC}}(q,\xi)=\frac{4\pi}{q\cdot V_{\mathrm{A}}}\int_0^{\infty} H(\xi-r)\cdot r^{D_{\mathrm{f}}-2}\cdot\sin(q\cdot r)dr=\frac{4\pi}{q\cdot V_{\mathrm{A}}}\int_0^{\xi} r^{D_{\mathrm{f}}-2}\cdot\sin(q\cdot r)dr \tag{S1}$$

Herein, $H(\xi-r)$ = 1 for $r < \xi$ and $H(\xi-r)$ = 0 for r > ξ. Since the volume or primary units was assumed infinite small $S(q)$ was being normalized over its entire agglomerate volume V_{A}.

Instead of using the unit step function we can also move the upper boundary of the sine transform from ∞ to ξ, as shown in the right hand side part of Equation (S1). Secondly, polydispersity is introduced by the integral:

$$S_{\mathrm{SC}}(q,\xi)=\int_0^\infty w(\xi)\cdot S_{\mathrm{HC}}(q,\xi)d\xi \tag{S2}$$

Herein, $w(\xi)$ is an intensity weighted probability density function of the cutoff parameter ξ. We applied a Schultz-Zimm distribution (Kotlarchyk & Chen, 1983), which was found to give realistic results in earlier studies on similar systems (Besselink et al., 2013; Stawski et al., 2011a,b; Pontoni et al., 2002):

$$w(\xi,\mu,z)=\frac{a^{Z+1}}{\Gamma(Z+1)}\cdot\xi^{Z}\cdot\exp(-a\cdot\xi) \tag{S3}$$

where

$$a=\frac{Z+1}{\mu}$$

and μ is the intensity weighted average of ξ and the Z-parameter is related to the distribution of the cutoff distance, i.e., the variance of ξ corresponds to $(\sigma_{\xi})^2 = \mu^2/(Z+1)$. By combining Equation (S1)-(S3) we obtain:

$$S_{\mathrm{SC}}(q,a,D_{\mathrm{f}},Z)=\frac{4\pi\cdot a^{Z+1}}{q\cdot V_{\mathrm{P}}\cdot\Gamma(Z+1)}\cdot\int_0^\infty\left[\xi^{Z}\left(\int_0^\xi r^{D_{\mathrm{f}}-2}\cdot\sin(q\cdot r)dr\right)\cdot\exp(-a\cdot\xi)\right]d\xi \tag{S4}$$

This integral can be evaluated as a Laplace transform and for integer values of Z. An analytical solution is given by

$$S_{\mathrm{SC}}(q,a,D_{\mathrm{f}},\zeta)=\frac{4\pi\cdot a^{Z+1}}{2\mathrm{i}\cdot q\cdot V_{\mathrm{P}}\cdot\Gamma(Z+1)}\cdot(-1)^{Z}\cdot\frac{d^{Z}}{da^{Z}}\left(\frac{1}{a}\cdot\left(\frac{1}{(a-\mathrm{i}\cdot q)^{D-1}}-\frac{1}{(a+\mathrm{i}\cdot q)^{D-1}}\right)\right) \tag{S5}$$

where i is the imaginary number. The derivatives of a that are expanding with increasing Z can be generalized by the following Riemann's sum:

$$S_{\mathrm{SC}}(q,a,D_{\mathrm{f}},\zeta)=\frac{4\pi}{2\mathrm{i}\cdot q\cdot V_{\mathrm{P}}}\cdot\sum_{\eta=0}^{Z}\left(\frac{\Gamma(D_{\mathrm{f}}+\eta-1)}{\Gamma(\eta+1)}\cdot a^{\eta}\cdot\left(\frac{1}{(a-\mathrm{i}\cdot q)^{D_{\mathrm{f}}+\eta-1}}-\frac{1}{(a+\mathrm{i}\cdot q)^{D_{\mathrm{f}}+\eta-1}}\right)\right) \tag{S6}$$

Here, η is an integer variable that varies from 0 to Z. In analogy with the mass fractal structure function with an exponential cutoff Equation (S2), the function is normalized over its agglomerate volume V_{A} such that $S(q\rightarrow 0) = 1$. The Porod volume of such agglomerate is described by:

$$V_{\mathrm{A}}=4\pi\cdot\left(\frac{a^{Z+1}}{\Gamma(Z+1)}\right)\cdot\int_{0}^{\infty}\xi^{Z}\left(\int_{0}^{\xi}r^{D_{\mathrm{f}}-3}\cdot r^{2}dr\right)\cdot\exp(-a\cdot\xi)d\xi \tag{S7}$$

which corresponds to:

$$V_{\mathrm{A}}=\frac{4\pi\cdot\Gamma(D_{\mathrm{f}}+Z+1)}{D_{\mathrm{f}}\cdot\Gamma(Z+1)}\cdot\left(\frac{\mu}{Z+1}\right)^{D_{\mathrm{f}}} \tag{S8}$$

Then, after normalization of Equation (S6) with Equation (S8), and replacing complex elements with goniometric equations we obtain:

$$S_{\mathrm{SC}}(q,a,D_{\mathrm{f}},Z)=\left(\frac{a}{q}\right)\cdot\frac{D_{\mathrm{f}}\cdot\Gamma(Z+1)}{\Gamma(D_{\mathrm{f}}+Z+1)}\cdot\sum_{\eta=0}^{Z}\left(\frac{\Gamma(D_{\mathrm{f}}+\eta-1)}{\Gamma(\eta+1)}\cdot\frac{\sin\left((D_{\mathrm{f}}+\eta-1)\cdot\mathrm{atan}\left(\frac{q}{a}\right)\right)}{\left(1+\left(\frac{q}{a}\right)^{2}\right)^{\left(\frac{(D_{\mathrm{f}}+\eta-1)}{2}\right)}}\right) \tag{S9}$$

Now, let us define ζ as the integer part of Z and ϕ as the fractional part of Z. Subsequently, we may approximate the structure function including fractional values as a linear combination of $S(\zeta)$ and $S(\zeta+1)$, i.e. $S(q) = (1-\phi)\cdot S(\zeta) + \phi\cdot S(\zeta+1)$. Since $S(\zeta+1) = S(\zeta) + S_{\mathrm{F}}(\zeta+1)$, where $S_{\mathrm{F}}(\zeta+1)$ only contains the $\zeta+1$ component of the Riemann's sum, this can be simplified to:

$$S_{\mathrm{SC}}(q,\mu,D_{\mathrm{f}},Z)=\frac{D_{\mathrm{f}}\cdot\Gamma(Z+2)}{(q\cdot\mu)\cdot\Gamma(D_{\mathrm{f}}+Z+1)}\cdot\left(S_{\mathrm{I}}+\phi\cdot S_{\mathrm{F}}\right) \tag{S10}$$

where:

$$S_{\mathrm{I}}=\sum_{\eta=0}^{\zeta}\left(\frac{\Gamma(D_{\mathrm{f}}+\eta-1)}{\Gamma(\eta+1)}\cdot\frac{\sin\left((D_{\mathrm{f}}+\eta-1)\cdot\operatorname{atan}\left(\frac{q\cdot\mu}{Z+1}\right)\right)}{\left(1+\left(\frac{q\cdot\mu}{Z+1}\right)^{2}\right)^{\left(\frac{(D_{\mathrm{f}}+\eta-1)}{2}\right)}}\right)$$

and:

$$S_{\mathrm{F}}=\frac{\Gamma(D_{\mathrm{f}}+\zeta)}{\Gamma(\zeta+2)}\cdot\frac{\sin\left((D_{\mathrm{f}}+\zeta)\cdot\operatorname{atan}\left(\frac{q\cdot\mu}{Z+1}\right)\right)}{\left(1+\left(\frac{q\cdot\mu}{Z+1}\right)^{2}\right)^{\left(\frac{(D_{\mathrm{f}}+\zeta)}{2}\right)}},$$

ζ = floor (Z) and $\phi = Z - \zeta$.

Here S_{I} and S_{F} represent the contributions of the integer and fractional values of Z to $S_{\mathrm{SC}}(q)$.

$$I(q)=I_0\cdot S(q) \tag{S11}$$

where $I_0 = N\cdot(V_A)^2\cdot(\Delta\rho)^2$, which corresponds to the scattering intensity at $q\to 0$ (since $S(q\to 0) = 1$), N is the particle number density, V_A the particle volume of the fractalic agglomerate (Equation (S8)) and $\Delta\rho$ and is the averaged difference in electron density between particles and their surroundings. For comparison of the Schultz cut-off model with the exponential cutoff model it is more convenient to express the size of a cluster by the radius of gyration that is derived from Feigin & Svergun (1987) and Porod (1982):

$$\left(R_{\mathrm{G}}\right)^{2}=\frac{1}{2}\cdot\frac{\int_{0}^{\infty} r^{4}\cdot\gamma(r)dr}{\int_{0}^{\infty} r^{2}\cdot\gamma(r)dr} \tag{S12}$$

which corresponds to:

$$\left(R_{\mathrm{G}}\right)^{2}=\frac{1}{2}\cdot\frac{\int_{0}^{\infty}\xi^{z}\left(\int_{0}^{\xi} r^{4}\cdot r^{D_{\mathrm{f}}-3}dr\right)\cdot\exp(-a\cdot\xi)d\xi}{\int_{0}^{\infty}\xi^{z}\left(\int_{0}^{\xi} r^{2}\cdot r^{D_{\mathrm{f}}-3}dr\right)\cdot\exp(-a\cdot\xi)d\xi} \tag{S13}$$

The solution is

$$R_{\mathrm{G}}=\left(\frac{\mu}{Z+1}\right)\cdot\sqrt{\frac{1}{2}\cdot\frac{D_{\mathrm{f}}\cdot(D_{\mathrm{f}}+Z+1)\cdot(D_{\mathrm{f}}+Z+2)}{(D_{\mathrm{f}}+2)}} \tag{S14}$$

Since $\gamma(r)$ is essentially an auto-convolution product of $\Delta\rho(r)$ a hard cutoff function is not realistic. The relative variance of ξ that can be derived from the Z parameter is always larger, because the relative variance of R_G and the relationship between Z and polydispersity depends on the geometry of the fractal. Alternatively, we may extract a polydispersity factor C_P following the procedure described by Sorensen and Wang (1999). Provided that $S(q)$ is normalized over the entire agglomerate volume Equation (S8), such that $S(q\rightarrow 0)=1$), the effective structure function in the fractal regime ($q\cdot R_G >> 1$) is described by:

$$S_{\mathrm{eff}}(q,R_{\mathrm{G}})=C\cdot C_{\mathrm{P}}\cdot(q\cdot R_{\mathrm{G}})^{-D_{\mathrm{f}}} \quad \text{for:} \quad q\cdot R_{\mathrm{G}}>>1 \tag{S15}$$

where the constant C is related to the geometry of the fractalic agglomerate and C_P is a measure of the polydispersity (Sorensen and Wang, 1999). Experimental data revealed that $C = 1.0 \pm 0.05$ for mass fractals with D_f between 1.7 and 2.1 (Sorensen and Wang, 1999).

The C_P value increases with increasing polydispersity and can be associated with a particular growth mode, i.e. C_P~1.5 for diffusion limited cluster aggregation (DLCA) and $C_P >$ 2 for reaction limited cluster aggregation (RLCA) (Sorensen and Wang, 1999). C_P is a size independent measure of polydispersity and depends solely on Z and D_f.. It can be derived from Equation (S9) by taking the limit $(q \cdot R_G) \rightarrow \infty$ of $S_{SC}(q \cdot R_G) \cdot (q \cdot R_G)^{D_f}$, which corresponds to:

$$C \cdot C_{\mathrm{P}} = \sin\left[(D_{\mathrm{f}} - 1) \cdot \frac{\pi}{2}\right] \cdot \left(\frac{D_{\mathrm{f}} \cdot \Gamma(D_{\mathrm{f}} - 1) \cdot \Gamma(Z+1)}{\Gamma(D_{\mathrm{f}} + Z + 1)}\right) \cdot \left(\frac{1}{2} \cdot \frac{D_{\mathrm{f}} \cdot (D_{\mathrm{f}} + Z + 1) \cdot (D_{\mathrm{f}} + Z + 2)}{(D_{\mathrm{f}} + 2)}\right)^{\left(\frac{D_{\mathrm{f}}}{2}\right)}$$

(S16)

Note that the Riemann's sum diminished since the limit was dominated by the $\eta = 0$ element of the Riemann's sum. As illustrated in Figure 2 by simulations of $S_{SC}(q,\mu,D_f,Z)$ with R_G =10 nm and D_f = 2, the height of the fractal regime as characterized by $C \cdot C_P$ decreases with increasing Z value.

2. References

Besselink, R., Stawski, T. M., Castricum, H. L. & ten Elshof, J. E. (2013). *Journal of Colloid and Interface Science* **404**, 24-35.

Feigin, L. A. & Svergun, D. U. (1987). *Struncture analysis by small angle X-ray and neutron scattering*. New York: Plenum Press.

Kotlarchyk, M. & Chen, S. H. (1983). *The Journal of Chemical Physics* **79**, 2461.

Pontoni, D., Narayanan, T. & Rennie, A. (2002). *Langmuir* **18**, 56-59.

Porod, G. (1982). *Small Angle X-ray Scattering*. New York: Acedamic Press.

Sorensen, C. M. & Wang, G. M. (1999). *Physical Review E - Statistical Physics, Plasmas, Fluids, and Related Interdisciplinary Topics* **60**, 7143-7148.

Stawski, T. M., Veldhuis, S. A., Besselink, R., Castricum, H. L., Portale, G., Blank, D. H. A. & Ten Elshof, J. E. (2011a). *Journal of Physical Chemistry C* **115**, 20449-20459.

Stawski, T. M., Veldhuis, S. A., Besselink, R., Castricum, H. L., Portale, G., Blank, D. H. A. & Ten Elshof, J. E. (2011b). *Journal of Physical Chemistry C* **115**, 24028.
